# Dermal Neutrophil, Macrophage and Dendritic Cell Responses to *Yersinia pestis* Transmitted by Fleas

**DOI:** 10.1371/journal.ppat.1004734

**Published:** 2015-03-17

**Authors:** Jeffrey G. Shannon, Christopher F. Bosio, B. Joseph Hinnebusch

**Affiliations:** Plague Section, Laboratory of Zoonotic Pathogens, Rocky Mountain Laboratories, National Institute of Allergy and Infectious Diseases, National Institutes of Health, Hamilton, Montana, United States of America; Stanford University School of Medicine, UNITED STATES

## Abstract

*Yersinia pestis*, the causative agent of plague, is typically transmitted by the bite of an infected flea. Many aspects of mammalian innate immune response early after *Y*. *pestis* infection remain poorly understood. A previous study by our lab showed that neutrophils are the most prominent cell type recruited to the injection site after intradermal needle inoculation of *Y*. *pestis*, suggesting that neutrophil interactions with *Y*. *pestis* may be important in bubonic plague pathogenesis. In the present study, we developed new tools allowing for intravital microscopy of *Y*. *pestis* in the dermis of an infected mouse after transmission by its natural route of infection, the bite of an infected flea. We found that uninfected flea bites typically induced minimal neutrophil recruitment. The magnitude of neutrophil response to flea-transmitted *Y*. *pestis* varied considerably and appeared to correspond to the number of bacteria deposited at the bite site. Macrophages migrated towards flea bite sites and interacted with small numbers of flea-transmitted bacteria. Consistent with a previous study, we observed minimal interaction between *Y*. *pestis* and dendritic cells; however, dendritic cells did consistently migrate towards flea bite sites containing *Y*. *pestis*. Interestingly, we often recovered viable *Y*. *pestis* from the draining lymph node (dLN) 1 h after flea feeding, indicating that the migration of bacteria from the dermis to the dLN may be more rapid than previously reported. Overall, the innate cellular host responses to flea-transmitted *Y*. *pestis* differed from and were more variable than responses to needle-inoculated bacteria. This work highlights the importance of studying the interactions between fleas, *Y*. *pestis* and the mammalian host to gain a better understanding of the early events in plague pathogenesis.

## Author Summary

Flea-borne transmission is central to the natural history of the plague bacillus *Yersinia pestis*, and infection within the context of flea feeding may affect the pathogenesis of bubonic plague. We analyzed the mammalian host response to *Y*. *pestis* in the skin immediately after transmission by its natural vector, the rat flea *Xenopsylla cheopis*, to observe differences relative to the response to needle-inoculated bacteria. Our results show that uninfected flea bites induce minimal inflammation, but flea-transmitted *Y*. *pestis* cause the recruitment of neutrophils roughly in proportion to the number of bacteria deposited in the skin. We observed interactions of flea-transmitted bacteria with macrophages, a cell type much more permissive than neutrophils for survival and growth of *Y*. *pestis*. We found that dendritic cells, important sentinel antigen presenting cells, were recruited to, but had minimal interaction with, flea-transmitted bacteria. Additionally, we found that *Y*. *pestis* could disseminate from the flea bite site to the draining lymph node and spleen as early as 1 h after flea feeding, significantly earlier than has been previously reported. This study reveals important differences between needle-inoculated and flea-transmitted *Y*. *pestis* in the immediate host response to infection and improves our understanding of the early host-bacterium interactions in plague pathogenesis.

## Introduction

Bubonic plague is the most common form of plague in humans and is the result of transmission of *Yersinia pestis* into the dermis via the bite of an infected flea. The bacteria survive in the skin and eventually disseminate to the dLN where they replicate to high numbers forming an enlarged lymph node termed a bubo. The cellular architecture of this bubo eventually becomes compromised resulting in hematogenous spread of the bacteria followed rapidly by death of the host. Fleas can also deposit bacteria directly into the bloodstream of a mammalian host resulting in primary septicemic plague that may constitute as many as one third of human cases [1,2].

*Y*. *pestis* evolved from its closest relative *Y*. *pseudotuberculosis*, approximately 1500 to 6400 years ago [3]. An essential step in evolution from an orally acquired pathogen that causes mild gastroenteritis to a highly pathogenic, flea-transmitted pathogen was aquistion of the ability to form a biofilm in the flea [4]. This biofilm blocks the proventriculus, a valve structure between the esophagus and midgut of the flea, and interferes with the flea’s ability to take a blood meal [5]. Blocked fleas make repeated attempts to feed until they eventually succumb to starvation or dehydration. Lorange *et al*. studied the vector efficiency of blocked rat fleas and found that less than half of the fleas transmitted *Y*. *pestis* while attempting to feed. For the fleas that did transmit, as many as 4000 CFU were detected, but the median number transmitted was 82 CFU [6].

The exact events that occur in the dermis immediately after deposition of *Y*. *pestis* by a flea remain enigmatic. Macrophages are considered permissive for *Y*. *pestis* survival whereas neutrophils are much more bactericidal toward the organism [7]; however, up to 10% of *Y*. *pestis* may survive after phagocytosis by neutrophils [8,9]. *Y*. *pestis* has been observed within macrophages and neutrophils early after intraperitoneal infection [10], but it is unclear if an intracellular phase is important in bubonic plague pathogenesis.

The most important virulence factor of *Y*. *pestis* is the pCD1 plasmid-encoded type III secretion system (T3SS). The T3SS effector proteins are preferentially translocated into phagocytes *in vivo* [11] where they disrupt multiple signaling pathways in phagocytes resulting in cellular paralysis, necrosis or apoptosis [12]. Genes encoding the T3SS are induced by growth at 37°C, but minimally expressed in the flea midgut [13, 14]. *Y*. *pestis* also produces a proteinaceous antiphagocytic capsule called F1. Similar to the T3SS, the F1 capsule is poorly expressed in the flea and induced by growth at 37°C [14,15]. Thus, there is likely a period immediately after deposition of the bacteria in the dermis until the T3SS apparatus, its secreted effectors and F1 capsule can be expressed when the *Y*. *pestis* is vulnerable to phagocytes.

Neutrophils are highly phagocytic innate immune cells that ingest and destroy invading bacteria. We have previously used intravital microscopy to examine *Y*. *pestis*-host cell interactions *in vivo* [16]. We found that large numbers of neutrophils are recruited to the infection site within 2–3h after i.d. injection of *Y*. *pestis*. Interestingly, recruited neutrophils rapidly associated with bacteria and many trafficked *Y*. *pestis* away from the injection site. In contrast, dendritic cells (DC), potent antigen presenting cells, were not recruited to the injection site and showed minimal interaction with bacteria [16].

Many previous studies of the early events following *Y*. *pestis* infection, including our own, have used intradermal needle inoculation to model bubonic plague transmission. However, needle inoculation differs from the natural route of transmission, the bite of an infected flea, in a number of ways. The flea mouthparts that are inserted into the skin during feeding are at least an order of magnitude smaller in diameter than the 30 gauge needle used for i.d. injections. Flea saliva also contains a number of molecules whose homologs in other blood feeding arthropods affect innate immunity [17]. Additionally, *Y*. *pestis* isolated from flea midguts display a markedly different phenotype when compared to *in vitro* cultured bacteria, including increased expression of biofilm extracellular matrix components and antiphagocytic factors [14]. Thus, we hypothesized that transmission by the natural plague vector might alter the numbers or composition of innate immune cells recruited to the site of infection and their interactions with bacteria. The low transmission efficiency of the flea vector makes quantitative assessment of cellular interactions difficult and led us to develop intravital microscopy methods to study these interactions *in vivo*. The goal of the present study was to characterize the *Y*. *pestis*-host cell interactions that occur in the dermis early after transmission of bacteria by the rat flea *Xenopsylla cheopis*.

## Results

### Identification of flea bite sites in the mouse dermis

**Fig 1:**
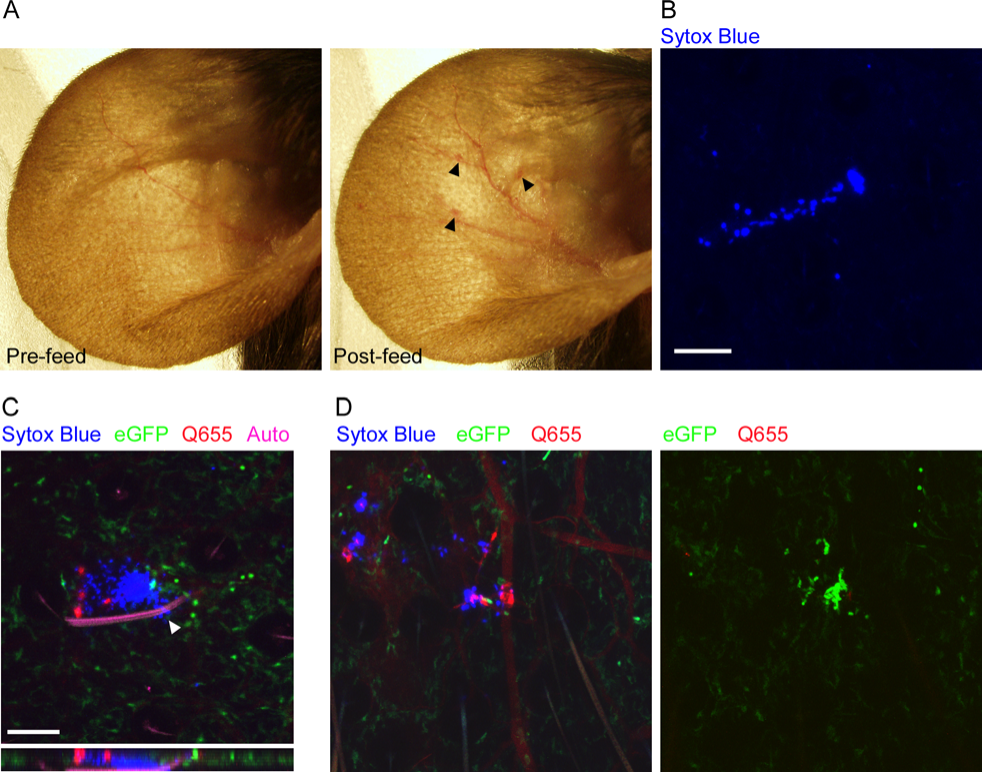
Characterization of flea bites in mouse skin. (A), dissecting microscope images of a mouse ear before and after being fed upon by 3 blocked fleas for 50 min. Arrowheads indicate small spots of erythema. (B), Confocal image of the ear of a mouse injected i.p. with Sytox Blue prior to being fed upon by an uninfected flea. The blue color indicates an area where cells have been damaged by flea feeding. (C) Confocal image of the ear of a LysM-eGFP mouse that was injected with Sytox Blue i.p. and Qtracker655 vascular dye i.v. prior to being fed upon by a blocked flea. During feeding the flea was anesthetized with isoflurane and its embedded mouthparts cut with microscissors. GFP^dim^ cells are macrophages, GFP^bright^ cells are neutrophils, red is the Q655 vascular dye, blue is the Sytox Blue, and magenta is autofluorescence of the flea mouthparts. The arrowhead indicates where the flea mouthparts pierce the skin. The lower panel is an x-z cross-section through the bite site. (D), Confocal images of a mouse ear prepared exactly as in (C) after being fed upon by 2 uninfected fleas. The 0 h and 4 h time points are shown. The full time series can be seen in S1 Video. Scale bars represent 100 μm.

Before we could characterize the responses to flea-transmitted *Y*. *pestis in vivo*, we needed to develop methods for reliably identifying flea bite sites on the mouse ear and characterize the neutrophil response to uninfected flea bites. To examine the gross effects of flea feeding on mouse skin, we constructed a simple clamp-on feeding chamber that would allow fleas to feed on a mouse ear (S1 Fig). The chamber containing fleas was placed on the ear for 50 min and dissecting microscope images of the ear were captured before and after feeding. Overall, the most noticeable effect of uninfected flea feeding was marked vasodilation in the ear (Fig. 1A). Occasionally, a flea bite would result in a small, discrete erythematous spot at the bite site, but more often there was no obvious visible indicator of where the fleas had fed. Similar results were seen after feeding of uninfected and infected fleas (S2 Fig). The absence of any consistent localized gross pathology after flea feeding made it difficult to reliably identify flea bite sites. Fortunately, intraperitoneal injection of mice with the non-membrane permeant fluorescent DNA stain Sytox Blue prior to flea feeding resulted in the staining of cells damaged as the fleas inserted their mouthparts into the skin. Foci of Sytox Blue stained nuclei of damaged cells can be seen in areas where fleas have fed (Fig. 1B). To confirm that these areas are flea bites, we injected mice i.v. with the vascular dye Q655 prior to flea exposure. Damage to blood vessels during flea feeding caused localized vascular dye leakage resulting in bright Q655 staining surrounding the vessel. These bright Q655 areas corresponded to areas of Sytox Blue staining (Fig. 1C, D left panel). As further confirmation of our ability to identify flea bite sites, we anesthetized fleas while their mouthparts were embedded in the mouse skin and used microscissors to cut the mouthparts off above the skin. The highly autofluorescent nature of the flea exoskeleton allowed for confocal imaging of the mouthparts, which were found embedded in an area containing both Sytox Blue and Q655 staining (Fig. 1C). Thus, the Sytox Blue method is a reliable way of identifying flea bite sites on mouse skin.

### Neutrophil responses to uninfected flea bites

**Fig 2:**
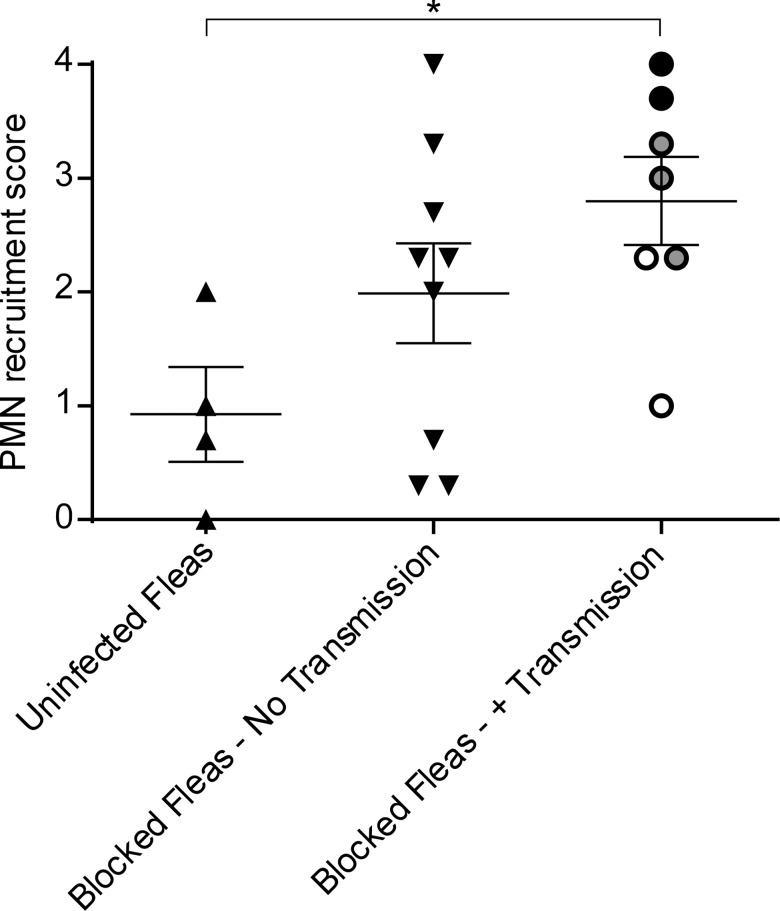
Scoring of neutrophil recruitment to uninfected flea bites, blocked flea bites without transmission and blocked flea bites that transmitted *Y*. *pestis*. LysM-eGFP mice that had been fed upon by uninfected fleas or infected fleas that were blocked with *Y*. *pestis* pMcherry were imaged by confocal and videos starting at ~15 min after flea feeding and going for ~4 h were captured. Each video was assigned a PMN recruitment score ranging from 0 (essentially no net neutrophil recruitment over the course of the experiment) to 4 (robust neutrophil accumulation resulting in formation of a large eGFP^bright^ cell aggregate at the flea bite site). The videos consisted of 3 categories, uninfected flea bites, blocked flea bites where no bacteria could be observed, and blocked flea bites where mCherry-expressing bacteria were apparent. For the Blocked Fleas- +Transmission column, the white, grey and black symbols correspond to experiments where low, moderate and high numbers of *Y*. *pestis* were present at the bite site, respectively. Horizontal bars on graph represent the mean, error bars represent SEM. * = p≤0.05.

To characterize the neutrophil response to individual uninfected flea bites, we fed fleas on LysM-eGFP transgenic mice that express high levels of eGFP in neutrophils and lower levels in macrophages in the skin [18]. The total neutrophil recruitment to the flea bite sites was evaluated and assigned a numerical score from 0 (no recruitment of neutrophils) to 4 (influx of large numbers of neutrophil resulting in a aggregated mass of eGFP^bright^ cells at the bite site). We found that uninfected flea feeding recruited remarkably few eGFP^bright^ neutrophils to the flea bite, despite the cellular damage and vascular leakage present at the bite site (identified by Sytox Blue or Q655 staining, respectively) (Fig. 1D, S1 Video). Neutrophil recruitment scores for four independent experiments ranged from 0 to 2 with a mean of 0.9 +/-0.4 (Fig. 2). In Fig. 1D multiple flea bites can be seen in the micrograph by Sytox Blue and Q655 staining. Neutrophils appear to be much more heavily recruited to the flea bite near the center of the field. Interestingly, we observed the mobilization and migration of eGFP^dim^ cells towards the bite site over the course of the experiment (S1 Video). Similar eGFP^dim^ cell movement towards uninfected flea bites was observed in four independent experiments. These eGFP^dim^ cells have been characterized as F4/80+, CD11b+ macrophages in the dermis of the LysM-eGFP mouse [18]. Thus, uninfected flea bites result in very little neutrophil recruitment and resident macrophages appear to migrate towards flea bite sites.

### Neutrophil responses to blocked flea bites without transmission of *Y*. *pestis*

**Fig 3:**
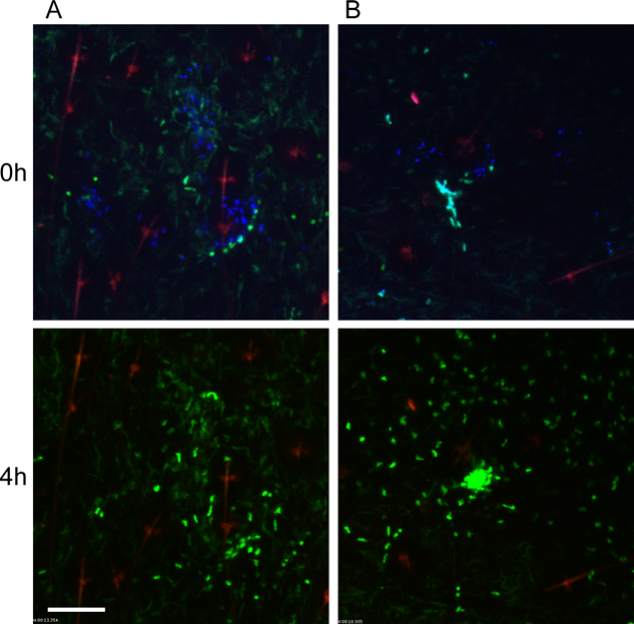
Responses of neutrophils and macrophages to blocked flea bites without transmission of *Y*. *pestis*. (A and B) two representative examples of confocal images of Lys-eGFP mouse ears after being fed upon for 50 min by *Y*. *pestis* pMcherry blocked fleas (left = 2 fleas, right = 4 fleas). Mice were injected with Sytox Blue i.p. prior to flea feeding. Upper panels show t = 0 h and lower panels show t = 4 h. The full time series can be seen in S2 and S3 Videos. Sytox Blue staining was used to identify the flea bite sites (blue, shown in upper panels only). GFP^dim^ cells are macrophages, GFP^bright^ cells are neutrophils, the red channel would have shown *Y*. *pestis* pMcherry if they were present. The red seen in these images is autofluorescent background of the hair and hair follicles. Scale bar represents 100 μm.

*Y*. *pestis* is typically transmitted by fleas in which the bacteria have established a biofilm that blocks the proventriculus. These blocked fleas are unable to take a normal blood meal and make repeated unsuccessful attempts to feed, partially withdrawing their mouthparts and reprobing. We hypothesized that this might result in more damage to the skin and consequently increased neutrophil recruitment in comparison to uninfected flea bites. To test this, blocked fleas infected with a T3SS deficient strain of *Y*. *pestis* expressing the fluorescent protein mCherry were allowed to feed on LysM-eGFP mice for approximately 50 min. Again, the Sytox Blue reagent was used to identify flea bite sites. We found obvious flea bites where bacteria could not be detected in >50% of the experiments involving feeding of blocked fleas, which is in agreement with what is known about flea transmission efficiency [6]. These bites were imaged to determine the neutrophil response to blocked flea bites without the influence of bacteria at the bite site. We observed a highly variable neutrophil response to blocked flea bites (Fig. 3). The responses ranged from recruitment of very few neutrophils (Fig. 3A, S2 Video), similar to what is seen with uninfected fleas, to an influx of large numbers of neutrophils to the flea bite site (Fig. 3B, S3 Video). When the neutrophil recruitment in nine independent experiments was scored, the results ranged from scores of 0 to 4 with a average score of 2 +/- 0.4 (Fig. 2). The skin of mice fed on by blocked fleas had more foci of Sytox Blue staining than skin fed on by uninfected fleas and these foci often appeared in clusters, presumably due to a flea making repeated attempts to feed in the same location. The numbers of neutrophils recruited did not appear to correlate with the amount of Sytox Blue staining at the bite site (Fig. 3). Thus, the neutrophil response to blocked flea bites is much more variable than the response to uninfected flea bites.

Similar to observations of uninfected flea bites, movement and migration of eGFP^dim^ macrophages towards blocked flea bites was common, occurring in seven out of nine independent experiments. In the two experiments where macrophage migration was not seen, large numbers of eGFP^bright^ neutrophils were recruited to the bite site, which may have obscured observation of the macrophage movement.

### Neutrophil responses to flea-transmitted *Y*. *pestis*

**Fig 4:**
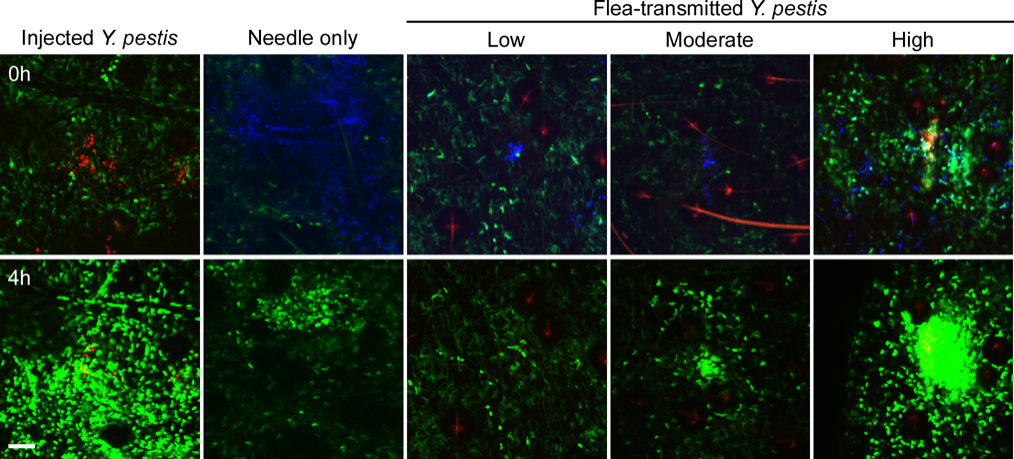
Imaging neutrophil and macrophage response to flea-transmitted *Y*. *pestis* in the dermis *in vivo*. Confocal images of Lys-eGFP mouse ears after being fed upon for 50 min by fleas infected and blocked with *Y*. *pestis* pMcherry (Low = 2 fleas, Moderate = 5 fleas, High = 2 fleas). Images of needle-inoculated *Y*. *pestis* expressing dsRed (~1000 CFU) (adapted from Shannon et al. [16]) and a sterile needle-stick site are shown for comparison. Mice were injected with Sytox Blue i.p. prior to flea feeding. Upper panels show t = 0 h and lower panels show t = 4 h. The full time series can be seen in S4–S6 Videos. Sytox Blue staining was used to identify the flea bite sites (blue, shown in upper panels only for all experiments except the needle-inoculated *Y*. *pestis*). GFP^dim^ cells are macrophages, GFP^bright^ cells are neutrophils, red is *Y*. *pestis* pMcherry (near the center of each image, in the vicinity of intense Sytox Blue staining). Left, center and right panels show low (~10 or fewer bacteria), moderate (~100s of bacteria) and high (~1000s of bacteria) levels of transmission, respectively. Scale bar represents 100 μm.

To characterize the neutrophil response to *Y*. *pestis* transmitted via the bite of an infected flea, blocked fleas infected with *Y*. *pestis* pMcherry were fed on a LysM-eGFP mice, flea bites were identified by Sytox Blue staining, and bite sites that contained mCherry+ bacteria were imaged. Consistent with previous studies [6], fleas transmitted a highly variable number of bacteria into the skin (determined qualitatively by image analysis). We show three example experiments representing responses to low (roughly ten or fewer), moderate (roughly hundreds) and high (roughly thousands) numbers of transmitted bacteria as determined by visual inspection of the bite site (Figs. 4, S4, S5 and S6 Video). For the purpose of comparison, images from the 0 h and 4 h timepoints after needle inoculation of *Y*. *pestis* (~1000 CFU) or a sterile 30 gauge needle stick alone are also shown in Fig. 4. In seven independent experiments where bacteria could be seen at flea bites, the neutrophil recruitment scores ranged from 0 to 4 with an average of 2.8 +/- 0.4 (Fig. 2). Overall, the number of neutrophils recruited to bite sites containing bacteria was higher than for uninfected flea bites or blocked flea bites without transmission. Furthermore, neutrophil recruitment appeared to correlate with the number of bacteria present at the bite site (Fig. 2).

**Fig 5:**
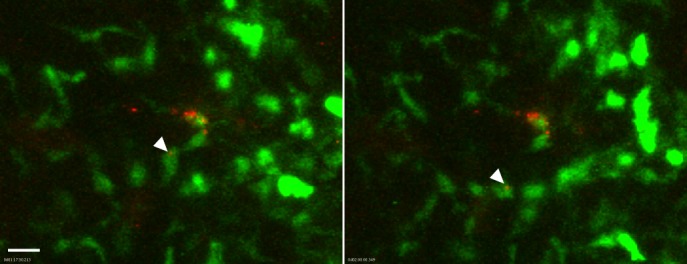
Association of flea-transmitted *Y*. *pestis* with eGFP^dim^ macrophages in the dermis *in vivo*. A Lys-eGFP mouse ear after being fed upon for 50 min by 5 *Y*. *pestis* pMcherry blocked fleas. Confocal images of ~1 h 17 min and 2 h time points (~1.5 h and 2.25 h post feeding, respectively) of a 4 h movie are shown. The full time series can be seen in S7 Video. The flea bite site was identified by Sytox Blue staining prior to acquisition of time series. Images were digitally magnified 6X from original. Arrowhead in each panel indicates *Y*. *pestis* (red) associated with the same GFP^dim^ macrophage (green) at each time point. Scale bar represents 20 μm.

Interestingly, even when large numbers of neutrophils were recruited to the bite site and associated with bacteria, very little translocation of the bacteria was observed. When bacteria were observed moving, they were frequently (observed in four out of seven experiments where bacteria were present at the flea bite site) associated with eGFP^dim^ cells, which are likely macrophages (Fig. 5, S7 Video). Movement of bacteria in association with eGFP^bright^ neutrophils was a rare event, observed in only 1 of the 7 experiments. This is in contrast to what is observed after needle inoculation of bacteria into the dermis, where many bacteria are trafficked away from the injection site in association with neutrophils [16].

Because the neutrophil recruitment to needle-inoculated *Y*. *pestis* is so robust, the presence of large numbers of eGFP^bright^ cells may obscure bacteria-eGFP^dim^ macrophage interactions. To address this possibility, we treated Lys-eGFP mice with anti-GR1, an antibody that efficiently depletes neutrophils and, to a lesser extent, inflammatory monocytes, thus permitting the visualization of the macrophage response to *Y*. *pestis* in the near absence of neutrophils. We observed movement of eGFP^dim^ macrophages towards the injection site (S8 Video), similar to what is seen in response to flea bites (S1–S5 Video). However, in contrast to what was observed after flea-transmission (S7 Video), in four independent experiments with needle-inoculated *Y*. *pestis* we did not observe movement of bacteria in association with eGFP^dim^ cells, suggesting that flea-transmitted *Y*. *pestis* may be more likely than needle-inoculated bacteria to interact with macrophages *in vivo*.

### Dendritic cell responses to flea-transmitted *Y*. *pestis*

Dendritic cells are antigen presenting cells that reside in peripheral tissues and migrate into the lymphatics after contact with pathogens [19]. To determine whether or not DC interact with *Y*. *pestis* after flea-borne transmission, we used a transgenic mouse expressing yellow fluorescent protein (YFP) under control of the *itgax* promoter [20]. *Itgax* encodes a component of CD11c, a molecule widely used to identify DCs.

**Fig 6:**
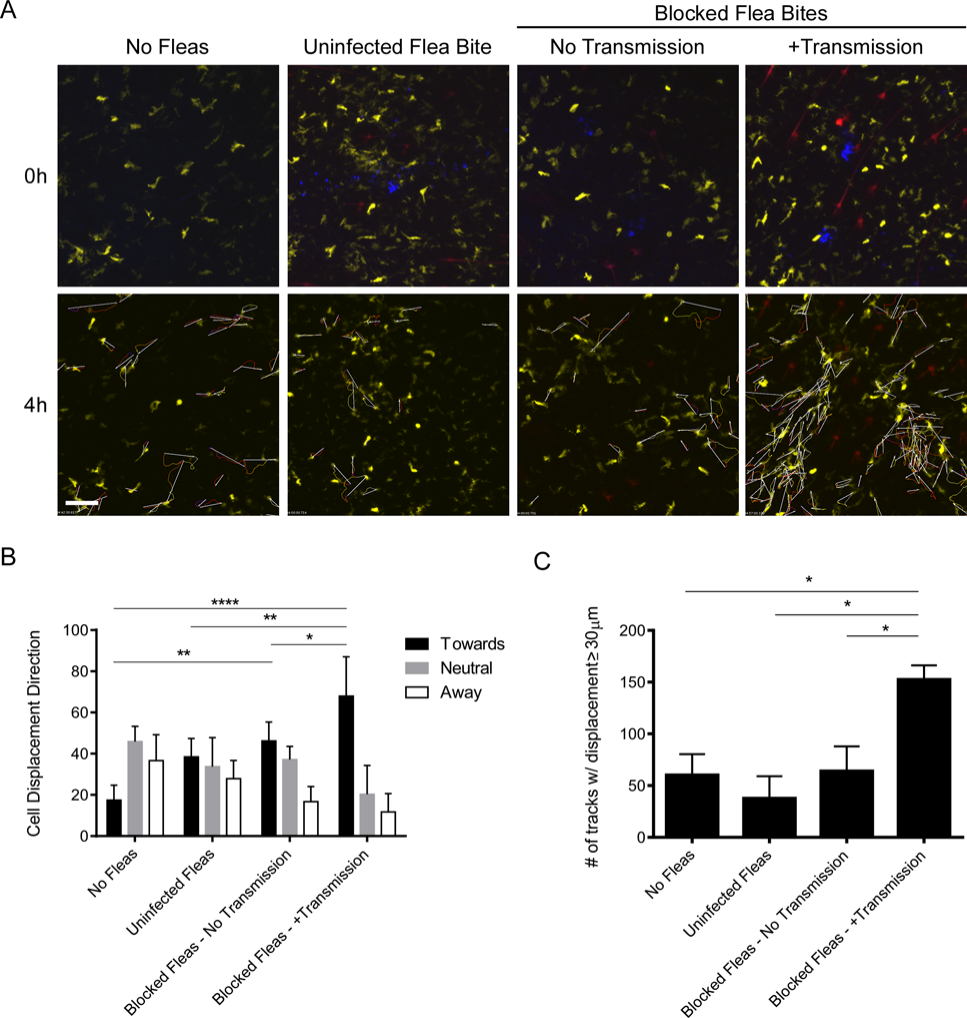
Imaging and tracking dendritic cell movement in response to flea bites and flea-transmitted *Y*. *pestis in vivo*. CD11c-YFP mouse ears were fed upon by 1 uninfected flea for 10 min, *Y*. *pestis* pMcherry blocked fleas for 50 min (No Transmission = 5 fleas, +Transmission = 6 fleas), or no fleas (empty feeding chamber) for 10 min and imaged by confocal microscopy for 4 h. (A) Flea bite sites were identified by Sytox Blue staining (blue, upper panels only). Upper panels show t = 0 h and lower panels show t = 4 h. The full time series can be seen in S9–S12 Videos. Movement of YFP^+^ over the course of the experiment was tracked using the image-processing software Imaris. The lower panels also show the cell tracking data and displacement (arrows) for all displacement events ≥ 30 μm. YFP^+^ cells are dendritic cells (yellow), the red channel shows *Y*. *pestis* pMcherry, if present. All examples shown are representative of at least 3 independent experiments. Scale bar represents 100 μm. (B) The direction and length of net displacement were calculated and analysis was limited to displacement events ≥ 30 μm. The direction of cell displacement was manually scored as being “toward,” “neutral,” or “away” from the flea bite site (determined by the presence of bacteria and/or Sytox Blue staining). For the No Fleas condition, a spot at the center of the image field was arbitrarily chosen as the reference point for the scoring. The results shown are the mean of at 3 or 4 independent experiments. Error bars represent standard deviation. (C), The average numbers of displacement events ≥ 30 μm in length for each experimental condition were calculated. Error bars represent SEM. The results shown are a mean of 3 or 4 independent experiments. * = p≤0.05, ** = p≤0.01, and **** = p≤0.0001.

The bite sites of uninfected fleas, blocked fleas that did not transmit bacteria, and blocked fleas that had deposited *Y*. *pestis* in the dermis were imaged for at least 4 hours post-feeding (Fig. 6A). In response to uninfected flea bites DCs appear to randomly move through the dermis (Fig. 6A, S9 Video), similar to what is observed in a naïve mouse ear (Fig. 6A, S10 Video). Consistent with what was observed after needle inoculation of these mice [16], we did not observe any notable interaction between DCs and flea-transmitted bacteria (Fig. 6A, S11 Video). Interestingly, while there was no net influx of a large number of DCs like that seen with neutrophils, the cells that were present appeared to migrate towards the flea bite sites that contained *Y*. *pestis*. A similar phenomenon was seen at some blocked flea bites where no bacteria were deposited in the dermis, but was much more variable (Fig. 6A, S12 Video). To quantify this cellular movement, image analysis software was used to track the migration of these cells over the course of the experiment. Cell tracking and displacement are shown in the bottom panels of Fig. 6A. The direction of displacement of each cell track was scored as being “toward”, “away” from or “neutral” relative to the flea bite site (Fig. 6B). Additionally, the average number of cell tracks with displacement >30 μm was determined for each experiment (Fig. 6C). We conclude that DCs migrate towards flea-transmitted *Y*. *pestis* or blocked flea bites, but not to uninfected flea bites in the dermis and that there was overall more displacement of DCs when bacteria were present at the bite site.

### *Y*. *pestis* CFU numbers in skin, draining lymph node and spleen after flea transmission

Fleas are considered to be primarily capillary feeders; they probe the skin with their mouthparts until a blood vessel is cannulated and a blood meal is siphoned directly from the vessel [21, 22]. Blocked fleas can deposit *Y*. *pestis* in the extravascular dermal tissue or, less frequently, directly into the lumen of a blood vessel [2]. To determine the numbers of bacteria transmitted by fleas in our experiments and the tissue localization of flea-transmitted *Y*. *pestis* early after infection, we collected ear dermis, dLN and spleen tissue samples from mice after completion of the intravital microscopy experiments described above (approximately 5 h after termination of flea feeding). Tissues were triturated and plated to determine the number of colony forming units (CFU) present. The results of each independent experiment consisting of an individual mouse are shown in S1 Table. The number of blocked fleas that fed on each mouse varied from one to seven. We recovered no CFUs from 22% of the mice tested despite many of these mice being fed upon by as many as four blocked fleas. Among mice that had detectable bacteria in the dermis after flea exposure, the number of dermal CFUs ranged from 5 to 3660 with a median of 237.5 CFUs.

The number of CFUs cultured from the spleen served as an indicator of transmission of bacteria directly into the bloodstream. Among the 28 mice that had detectable CFUs in any of the tissues tested, 23 (82%) had anywhere from 1 to 4000 CFUs/spleen. Two mice had bacteria in their spleens, but no bacteria were detected in their dermis or dLN, indicating that fleas had deposited bacteria directly into the lumen of blood vessel during the feeding attempt. Despite harvesting the tissues at the early time point of ~5 h post-feeding and the use of a highly attenuated strain of *Y*. *pestis*, we found a surprisingly high number of bacteria present in the dLN. The numbers ranged from 15 to 1000 CFUs with a median of 270 CFUs/LN. Thus, dissemination of bacteria from the dermis to draining lymph node can occur within 5 h of flea feeding.

**Fig 7:**
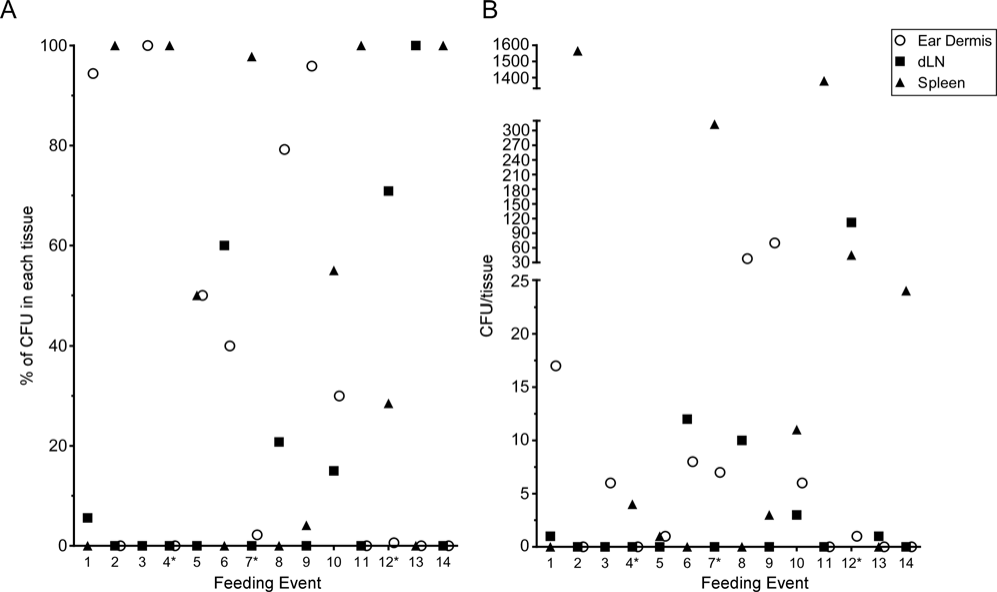
CFU transmission data at 1 h post-feeding from 31 individual blocked flea feeding events. Between 1 and 3 blocked fleas were exposed to the each ear of individual C57Bl/6 mice for 1 h. Mice that had at least one flea feed on one ear were euthanized 1 h post-flea exposure. The ears, ipsilateral mandibular and parotid lymph nodes and spleen were harvested, triturated and plated to determine *Y*. *pestis* CFU counts. The results of each individual feeding where bacteria were found in at least one tissue are shown. An * indicates a mouse with feeding events on each ear, thus CFU numbers in the spleen could reflect transmission in either or both ears. (A) the CFU data are shown as a percentage of the total CFU recovered from each tissue. (B) the raw CFU numbers found in each tissue are shown.

The above experiments were done in conjunction with the intravital microscopy studies, thus the mice were exposed to variable numbers of blocked fleas, received variable numbers of flea bites, and were assayed ~5 h after flea feeding. To quantify transmission by individual fleas, we performed experiments where mice were exposed to 1 to 3 blocked fleas placed on one or both ears in an effort to consistently obtain mice that had been fed on by only 1 blocked flea. Mice were euthanized 1 h after termination of flea exposure and their ear, dLN and spleen tissues assayed for CFU. Each time a mouse ear had been fed on by at least one blocked flea it was recorded as a feeding event. In total, we exposed a total of 25 mice and recorded 31 feeding events. Of these events, 14 (45.2%) resulted in transmission of bacteria into at least one of the tissues assayed and these are depicted in Fig. 7. Nine (29%) of the feeding events resulted in deposition of *Y*. *pestis* into the dermis. We detected bacteria in the spleen of 9 (29%) mice 1 h after removal of fleas, suggesting that bacteria were introduced directly into the bloodstream during flea feeding. Bacteria were detected in the dLN after 6 (19.4%) individual feeding events. The presence of bacteria in the dLN at this early time point indicates that some bacteria disseminate to the dLN within 2 h after introduction into the dermis.

## Discussion

The degree to which flea transmission influences the pathogenesis of bubonic plague or the innate immune response to infection is unknown. Here we characterize the very early neutrophil, macrophage and dendritic cell recruitment to flea bites and flea-transmitted *Y*. *pestis*. We developed a method for reliably and accurately identifying flea bite sites in mice using the DNA stain Sytox Blue. Using this method we imaged the host cellular response to uninfected flea bites and found minimal recruitment of neutrophils to the bite site. This was surprising in light of previous work showing a rapid neutrophil response to tissue damage [18, 23]. Specifically, a study by Peters et al. showed robust neutrophil recruitment to uninfected sand fly bites on the same LysM-eGFP mouse strain used in our study [18]. Sand flies are “pool feeders” in that they feed by wounding the dermal microvasculature with serrated mouthparts and siphoning blood from a hemorrhagic pool formed within the wound. In contrast, fleas are considered “capillary feeders” that use their small mouthparts to cannulate a dermal blood vessel, with apparently little damage to the cells at the bite site. This may result in less inflammation and neutrophil recruitment than is seen at sand fly bite sites. Additionally, flea saliva contains a variety of components homologous or analogous to salivary proteins in other blood feeding arthropods that are known to be anti-inflammatory [17]. Both of these factors may be responsible for the low numbers of neutrophils recruited to uninfected flea bites.

Interestingly, we observed the mobilization and migration of eGFP^dim^ cells towards the flea bite site. In the LysM-eGFP transgenic mouse used in this study, these eGFP^dim^ cells in the dermis are largely F4/80^+^ tissue resident macrophages [18]. We did not observe movement of these cells towards needle inoculation sites in our previous study [16], but the large numbers of eGFP^bright^ neutrophils recruited to tissue damage done by the needle made it difficult to see the dim macrophages.

When we examined blocked flea bites in LysM-eGFP mice where bacteria had been deposited at the bite site, we observed more neutrophil recruitment relative to uninfected flea bites and the neutrophil numbers appeared to correlate with the amount of bacteria at the site. This suggests that the neutrophils were recruited to the bacteria and not the bite itself. It also shows that any suppressive effect that flea saliva may have on neutrophil recruitment could not override the response to bacteria in the dermis. Despite the presence of a large number of neutrophils, we observed very little movement of *Y*. *pestis* at the bite site. This is in contrast to our previous study showing many injected bacteria being trafficked away from the injection site in association with eGFP^bright^ neutrophils [16].

*Y*. *pestis* isolated from flea midguts are more resistant to phagocytosis by macrophages and neutrophils than broth-cultured bacteria due to upregulation of a family of insecticidal-like toxin complex proteins in the flea [14, 24]. The two-component regulatory system PhoP-PhoQ, important for the resistance of *Y*. *pestis* to stressors experienced in the mammalian host such as low pH, osmotic or oxidative stress, or antimicrobial peptides is also upregulated in the flea relative to *in vitro* broth-cultured bacteria [14, 25]. Additionally, *Y*. *pestis* forms a biofilm in the flea midgut as result of increased production of a polysaccharide extracellular matrix (ECM) in this environment. The effects of this ECM on mammalian host response are unknown, but structurally similar ECM produced by *Staphylococci* protects against innate immune effectors [26]. Thus, the phenotype of flea-derived *Y*. *pestis* differs considerably from broth-grown bacteria in ways that may influence pathogenesis and innate host response. Further work will be needed to evaluate the interactions of flea-grown *Y*. *pestis* with innate immune cells *in vivo*.

Interestingly, when bacterial movement at the flea bite site was observed, many of these bacteria were associated with eGFP^dim^ macrophages. In each case the macrophages did not transport bacteria completely away from the injection site, but remained in the field of view for the duration of the experiment (Fig. 5, S7 Video). Again, this is in contrast to experiments with needle-inoculated bacteria where most of the *Y*. *pestis* movement was in association with neutrophils that transported bacteria completely out of the field of view [16]. However, the large number of eGFP^bright^ neutrophils present may have obscured the rare eGFP^dim^ events in these experiments. To address this, we needle-inoculated PMN-depleted LysM-eGFP mice with *Y*. *pestis* expressing dsRed and imaged them by confocal. While eGFP^dim^ macrophages were recruited to the injection site, we observed minimal movement of bacteria in association with these cells. These results suggest that flea-transmitted bacteria may preferentially interact with macrophages over neutrophils. This would have implications for *Y*. *pestis* pathogenesis, as macrophages are much more permissive for *Y*. *pestis* survival and growth than neutrophils [7].

Imaging of blocked flea bites in CD11c-YFP mice revealed minimal interactions between YFP^+^ cells and flea-transmitted *Y*. *pestis* at the bite site. It is important to note that YFP expression in these mice is not limited exclusively to DCs, nor does every subset of DC in the dermis express YFP. It is more accurate to classify the YFP^+^ dermal cells in our experiments as antigen-presenting mononuclear phagocytes [27]; however, for simplicity, we refer to these YFP^+^ cells as DCs. While we did not observe a massive influx of DCs, they did appear to mobilize and migrate specifically towards blocked flea bites containing bacteria. The consequences of this migration of DCs towards flea-transmitted bacteria are unknown. DCs do not appear to associate with bacteria at the bite site in the timeframe studied, but it remains possible that they would show more association with bacteria later after infection. These results are consistent with our previous study showing minimal interaction between needle inoculated *Y*. *pestis* and DC early after infection [16].

Uninfected flea bites did not recruit DCs and migration towards blocked flea bites where bacteria were not detected was variable. It is plausible that some bacterial components, such as lipopolysaccharide, could have been introduced into the bite site by blocked fleas even if no whole bacteria were transmitted. These bacterial components could act as pathogen associated molecular patterns (PAMPs) that directly or indirectly stimulate recruitment of innate immune cells [28]. It is also possible that a very small number of bacteria might have been transmitted, but were undetectable by microscopy. These factors may explain the variability in cellular response we observed. Additionally, blocked fleas are unable to take a blood meal and tend to probe the skin in repeated unsuccessful feeding attempts. The additional tissue damage from this probing could explain the increased cellular recruitment to blocked compared to uninfected flea bites, although the amount of Sytox Blue staining at the bite site did not appear to correlate with neutrophil recruitment.

The CFU assays of the dermis, dLN, and spleen early after flea feeding yielded highly variable results, consistent with previous studies on the regurgitative transmission mechanism of *X*. *cheopis* [6]. It was not uncommon to find several hundred or even thousands of bacteria in the spleen after flea feeding. This is most likely due to regurgitation of bacteria directly into the bloodstream during the blocked flea’s attempt to feed as has been previously described [2]. Interestingly, several animals had hundreds or more CFU in the dLN at ~5 h post-feeding. This prompted us to look 1 h post-feeding where we found some animals with a small number of CFU in the dLN. Overall, these data suggest that very rapid dissemination to the spleen and dLN is a common occurrence after flea transmission of *Y*. *pestis*. The data also suggest that a small number of flea-transmitted bacteria may move so rapidly into the lymphatics that they bypass any significant interactions with phagocytes at the bite site. The ultimate fate of these early LN disseminators is unknown.

Despite the historical significance of *Y*. *pestis* and the importance of fleas in the plague transmission cycle, the early events in the skin after deposition of bacteria via blocked flea bite are poorly understood. Here we have characterized the innate cellular recruitment to uninfected and infected flea bites *in vivo*. We also gathered quantitative data on the numbers and tissue distribution of *Y*. *pestis* transmitted by fleas. Our results show a much greater neutrophil response to flea-transmitted *Y*. *pestis* than to uninfected flea bites. We also observed migration of resident tissue macrophages towards uninfected and blocked flea bite sites and their association with flea-transmitted *Y*. *pestis*. Similar migration of dendritic cells towards infected, but not uninfected, flea bites was observed. Interestingly, we found *Y*. *pestis* in the dLN by 1 h after flea exposure, suggesting that initial dissemination of bacteria to the LN occurs more quickly than was previously appreciated [29, 30]. In support of this, Gonzalez *et al*. recently reported that needle-injected *Y*. *pestis* could be found in the dLNs of some mice as early as 10 min post-infection [31]. Future work will be aimed at determining the fate of these early disseminators and their importance in bubonic plague pathogenesis.

## Materials and Methods

### Flea infection

*Xenopsylla cheopis* fleas were infected with *Y*. *pestis* pMcherry (strain KIM6+ [virulence plasmid negative, pigmentation locus positive] transformed with a pMcherry plasmid [Clontech]) using a previously described artificial feeding system [5]. Fleas were monitored for blockage by microscopic examination for up to six weeks post-infection. Blockage was diagnosed by the presence of fresh blood in the flea esophagus, but not the midgut, immediately after feeding.

### Mice

C57BL/6J LysM-eGFP knock-in mice were originally created by T. Graf [32] (Albert Einstein University, Bronx, NY) and were bred by Taconic Laboratories under a contract with NIAID. C57BL/6J (stock number 000664) and CD11c-YFP (stock number 008829, originally described Lindquist *et al*. [20]) mice were purchased from The Jackson Laboratory (Bar Harbor, ME). Ten- To 20-week-old female mice were used in all experiments. All mice were maintained at the Rocky Mountain Laboratories animal care facility under specific-pathogen-free conditions.

For experiments involving PMN-depletion, mice were injected i.p. with 250 μg anti-GR1 antibody (clone RB6–8C5, BioXCell, West Lebanon, NH) 24 h and 4 h prior to infection with ~1000 CFU of dsRed-expressing *Y*. *pestis*, as described in [16]. This treatment results in >95% depletion of Ly6G^+^ neutrophils. The *Y*. *pestis* strain expressing dsRed was used instead of the mCherry-expressing strain in this experiment to be consistent with a previous study of the response to needle-inoculated *Y*. *pestis*, and due to a higher level of fluorescent protein expression in broth culture.

### Feeding fleas on mice

Mice were anesthetized by subcutaneous injection of a ketamine/xylazine mixture and secured on a heating pad to maintain body temperature. Where indicated, mice were injected with 250 μM Sytox Blue (Life Technologies) in 150 μL PBS i.p. and, in some cases, 60 μl of Qtracker655 (2 μM stock, Life Technologies) in 150 μl PBS i.v. 10 to15 min prior to flea exposure. Fleas were immobilized by incubation on ice and placed in a custom-made feeding chamber consisting of a 200 μl PCR tube and a foam padded plastic clamp (S1 Fig). This chamber was then clamped onto the ear of the mouse and the fleas allowed to warm to room temperature. The fleas were in contact with the mouse for 10 min for uninfected fleas or 50 min for blocked fleas. The mouse and feeding chamber were then placed in a jar containing isoflurane for approximately 30 sec to anesthetize the fleas. The chamber was then removed from the ear and the fleas collected and microscopically examined to determine if fresh blood was present in their digestive tract indicating feeding. In some cases, a model SMZ1500 dissecting microscope (Nikon, Tokyo, Japan) equipped with a model DP72 color camera (Olympus, Center Valley, PA) was used to capture images of mouse ears before and after exposure to fleas.

### Intravital microscopy

The ears of LysM-eGFP or CD11c-YFP mice were imaged by confocal microscopy as previously described [16]. Briefly, mice were anesthetized with an isoflurane-O_2_ mixture provide by nose cone and their ears mounted to a coverslip on the stage of a Zeiss LSM 510 Meta confocal microscope (Zeiss, Oberkochen, Germany) equipped with an incubated chamber set to 30°C. Z stacks were acquired with a 20x objective at 2 min intervals for the indicated duration and the images obtained were processed using Imaris 6.3.1 software (Bitplane, South Windsor, CT). All supplemental video files are shown at the same magnification with the exception of S7 Video which has been digitally zoomed using the Imaris software.

Neutrophil recruitment was scored by assessment of total neutrophil accumulation observed over the duration of videos of Lys-eGFP mice fed upon by uninfected or blocked fleas. Each video was scored by 3 lab members on a scale from 0 to 4 in whole number increments, with 0 representing essentially no net recruitment of neutrophils and 4 representing massive accumulation of neutrophils forming a large aggregate at the bite site. The results are shown as the mean and standard error of the mean (SEM).

Tracking of YFP^+^ cells in time series of CD11c-YFP mice was accomplished using the tracking function within the Imaris 6.3.1 software package. Once the cellular movement had been tracked, we used the software to determine the direction of net displacement of each cell. Limiting further analysis to YFP^+^ cells with a net displacement of at least 30 μm over the course of the experiment, we scored each cell displacement event as being towards (displacement within a 45° angle in the direction of the bite site), away (displacement within a 45° angle in the opposite direction of the bite site), or neutral (all remaining displacement events) relative to the flea bite site. For mice that had not been fed on by fleas, a spot at the center of field of view was arbitrarily chosen to represent the flea bite site.

### Tissue colony forming unit (CFU) assay

Ear, draining cervical lymph node and spleen tissues were collected from mice after euthanasia. Ears were separated with forceps into ventral and dorsal halves. Tissues were placed in Lysing Matrix H bead tubes (MP Biomedicals) containing 500 μl of PBS and disrupted with a Fastprep 120 (Thermo Savant). The numbers of *Y*. *pestis* pMcherry CFU in the tissue samples were determined by dilution and plating on blood agar plates containing 100 μg/ml carbenicillin.

### Ethics

All animal studies were performed under protocols adhering to guidelines established by the Public Health Service Policy on Humane Care and Use of Laboratory Animals. The protocols were reviewed and approved by the Rocky Mountain Laboratories Animal Care and Use Committee (AALAS unit number 000462, PHS-OLAW number A-4149–01).

### Statistics

For experiments measuring neutrophil recruitment scores, data were analyzed using a Kruskal-Wallis nonparametric test followed by a Dunn’s multiple comparison test. For experiments determining the direction of DC migration, data were analyzed using two-way ANOVA with Tukey’s multiple comparison post-test. For experiments measuring total DC displacement, data were analyzed using one-way ANOVA with Holm-Sidak’s multiple comparisons post-test.

## Supporting Information

S1 Table CFU transmission data acquired after intravital microscopy experiments at ~5 h post-feeding. The indicated number of *Y*. *pestis* pMcherry blocked fleas were exposed to the right ear of individual LysM-eGFP or CD11c-YFP mice for 50 min. These mice were then imaged by intravital microscopy for approx. 4 h. Mice were euthanized at ~5 h post-feeding and the right ear, right mandibular and parotid lymph nodes, and spleen were harvested, triturated and plated to determine *Y*. *pestis* CFU counts. The results of each individual experiment are shown. Data are sorted in descending order based on the total number of fleas that fed on each mouse.
(DOCX)

S1 Fig Photographs of custom-made flea feeding chamber and confocal imaging setup. (TIFF)

S2 Fig Characterization of flea bites in mouse skin. Dissecting microscope images of a mouse ear before (Pre) and after (Post) being fed upon by 3 uninfected fleas for 10 min. No obvious flea bite sites can be seen, but blood vessel dilation in response to uninfected flea feeding is apparent.
(TIFF)

S1 Video Response to uninfected flea bites in LysM-eGFP mice. Time series of confocal imaging starting ~15 min after a LysM-eGFP mouse was fed upon by 2 uninfected fleas. Confocal Z stacks were collected every 2 min for 4 h. Prior to flea feeding the mouse was injected i.v. with Qtracker655 vascular dye (red) and i.p. with Sytox Blue (blue, shown only in first few seconds of movie) to allow identification of flea bites. eGFP^dim^ cells are macrophages, eGFP^bright^ cells are neutrophils. White arrowheads indicate 3 example eGFP^dim^ macrophages that mobilize and migrate towards the flea bite site. The fading of the Qtracker655 dye that has leaked from the blood vessel at the bite site is likely due to passive diffusion of the dye away from the damaged vessel.
(MP4)

S2 Video Response to blocked flea bites where *Y*. *pestis* was not transmitted (weak neutrophil recruitment [mean recruitment score = 0.7). Time series of confocal imaging starting ~15 min after a LysM-eGFP mouse was fed upon by 2 *Y*. *pestis* pMcherry blocked fleas. Confocal Z stacks were collected every 2 min for 4 h. Prior to flea feeding the mouse was injected i.p. with Sytox Blue (blue, shown only in first frame of movie) to allow identification of flea bites. eGFP^dim^ cells are macrophages, eGFP^bright^ cells are neutrophils. The red channel would show *Y*. *pestis* pMcherry if any were present. White arrowheads indicate 3 example eGFP^dim^ macrophages that mobilize and migrate in response to the flea bite.
(MP4)

S3 Video Response to blocked flea bites where *Y*. *pestis* was not transmitted (strong neutrophil recruitment [mean recruitment score = 3.3]). Time series of confocal imaging starting ~15 min after a LysM-eGFP mouse was fed upon by 4 *Y*. *pestis* pMcherry blocked fleas. Confocal Z stacks were collected every 2 min for 4 h. Prior to flea feeding the mouse was injected i.p. with Sytox Blue (blue, shown only in first few seconds of movie) to allow identification of flea bites. eGFP^dim^ cells are macrophages, eGFP^bright^ cells are neutrophils. The red channel would show *Y*. *pestis* pMcherry if any were present.
(MP4)

S4 Video An example of “low” numbers of flea-transmitted *Y*. *pestis* in LysM-eGFP mice. Time series of confocal imaging starting ~15 min after a LysM-eGFP mouse was fed upon by 2 *Y*. *pestis* pMcherry blocked fleas. Confocal Z stacks were collected every 2 min for 4 h. Prior to flea feeding the mouse was injected i.p. with Sytox Blue (blue, shown only in first few seconds of movie) to allow identification of flea bites. eGFP^dim^ cells are macrophages, eGFP^bright^ cells are neutrophils. *Y*. *pestis* pMcherry appear in red. Area containing bacteria indicated with arrows for first few seconds of movie. This video was scored for neutrophil recruitment and given a mean score of 1.0.
(MP4)

S5 Video An example of “moderate” numbers of flea-transmitted *Y*. *pestis* in LysM-eGFP mice. Time series of confocal imaging starting ~15 min after a LysM-eGFP mouse was fed upon by 5 *Y*. *pestis* pMcherry blocked fleas. Confocal Z stacks were collected every 2 min for 4 h. Prior to flea feeding the mouse was injected i.p. with Sytox Blue (blue, shown only in first few seconds of movie) to allow identification of flea bites. eGFP^dim^ cells are macrophages, eGFP^bright^ cells are neutrophils. *Y*. *pestis* pMcherry appear in red. This video was scored for neutrophil recruitment and given a mean score of 3.0.
(MP4)

S6 Video An example of “high” numbers of flea-transmitted *Y*. *pestis* in LysM-eGFP mice. Time series of confocal imaging starting ~15 min after a LysM-eGFP mouse was fed upon by 2 *Y*. *pestis* pMcherry blocked fleas. Confocal Z stacks were collected every 2 min for 4 h. Prior to flea feeding mouse was injected i.p. with Sytox Blue (blue, shown only in first few seconds of movie) to allow identification of flea bites. eGFP^dim^ cells are macrophages, eGFP^bright^ cells are neutrophils. *Y*. *pestis* pMcherry appear in red. This video was scored for neutrophil recruitment and given a mean score of 4.0.
(MP4)

S7 Video Digitally zoomed movie of flea-transmitted *Y*. *pestis* interaction with a eGFP^dim^ cell *in vivo*. Time series of confocal imaging starting ~15 min after a LysM-eGFP mouse was fed upon by 5 *Y*. *pestis* pMcherry blocked fleas. Confocal Z stacks were collected every 2 min for 4 h. Images have been digitally zoomed 6X focusing on bacteria. eGFP^dim^ cells are macrophages, eGFP^bright^ cells are neutrophils. *Y*. *pestis* pMcherry appear in red. Red bacteria associated with a eGFP^dim^ cell are indicated with an arrow at several time points throughout the movie.
(MP4)

S8 Video Imaging eGFP^dim^ macrophage movement in PMN-depleted LysM-eGFP mice. A LysM-eGFP mouse was injected i.p. 24 h and 4 h prior to infection with 250 μg anti-GR1 antibody to deplete neutrophils and injected i.d. with ~1000 *Y*. *pestis* expressing dsRed. Confocal Z stacks were collected every 2 min for 4 h. eGFP^dim^ cells are macrophages, eGFP^bright^ cells are neutrophils. The bacteria are red. Due to the depletion of neutrophils, very few eGFP^bright^ cells are recruited, allowing for visualization of the eGFP^dim^ macrophages. Video is representative of 4 independent experiments.
(MP4)

S9 Video Dendritic cell response to an uninfected flea bite. Time series of confocal imaging starting ~15 min after a CD11c-YFP mouse was fed upon by 1 uninfected flea. Confocal Z stacks were collected every 2 min for 4 h. Prior to flea feeding the mouse was injected i.p. with Sytox Blue (blue, shown only in first few seconds of movie) to allow identification of flea bites. YFP^+^ cells are shown in yellow.
(MP4)

S10 Video Dendritic cell responses in a naïve CD11c-YFP mouse that has not been exposed to fleas. Time series of confocal imaging starting ~15 min after an empty flea feeding chamber was placed on the ear of a CD11c-YFP mouse. Confocal Z stacks were collected every 2 min for 4 h. Prior to flea feeding the mouse was injected i.p. with Sytox Blue (blue, shown only in first few seconds of movie) to show that there were no flea bites or damage to the skin. YFP^+^ cells are shown in yellow.
(MP4)

S11 Video Dendritic cell response to a blocked flea bite where transmission of Y. pestis pMcherry occurred. Time series of confocal imaging starting ~15 min after a CD11c-YFP mouse was fed upon by 6 *Y*. *pestis* pMcherry blocked fleas. Confocal Z stacks were collected every 2 min for 4 h. Prior to flea feeding the mouse was injected i.p. with Sytox Blue (blue, shown only in first few seconds of movie) to allow identification of flea bites. YFP^+^ cells are shown in yellow. Bacteria are in red.
(MP4)

S12 Video Dendritic cell response to blocked flea bites where *Y*. *pestis* was not transmitted. Time series of confocal imaging starting ~15 min after a CD11c-YFP mouse was fed upon by 5 *Y*. *pestis* pMcherry blocked fleas. Confocal Z stacks were collected every 2 min for 4 h. Prior to flea feeding the mouse was injected i.p. with Sytox Blue (blue, shown only in first few seconds of movie) to allow identification of flea bites. YFP^+^ cells are shown in yellow. Bacteria, if they had been present, would be red.
(MP4)

## References

[1] Butler T. Plague gives surprises in the first decade of the 21st century in the United States and worldwide. Am J Trop Med Hyg. 2013;89[4]:788–93. Epub 2013/09/18. 10.4269/ajtmh.13-0191 24043686 PMC3795114

[2] Sebbane F, Jarrett CO, Gardner D, Long D, Hinnebusch BJ. Role of the *Yersinia pestis* plasminogen activator in the incidence of distinct septicemic and bubonic forms of flea-borne plague. Proc Natl Acad Sci USA. 2006;103[14]:5526–30. 16567636 10.1073/pnas.0509544103 PMC1414629

[3] Cui Y, Yu C, Yan Y, Li D, Li Y, Jombart T, et al Historical variations in mutation rate in an epidemic pathogen, *Yersinia pestis* . Proc Natl Acad Sci U S A. 2013;110[2]:577–82. Epub 2012/12/29. 10.1073/pnas.1205750110 23271803 PMC3545753

[4] Chouikha I, Hinnebusch BJ. *Yersinia*-flea interactions and the evolution of the arthropod-borne transmission route of plague. Current opinion in microbiology. 2012;15[3]:239–46. Epub 2012/03/13. 10.1016/j.mib.2012.02.003 22406208 PMC3386424

[5] Hinnebusch BJ, Perry RD, Schwan TG. Role of the *Yersinia pestis* hemin storage (*hms*) locus in the transmission of plague by fleas. Science. 1996;273(5273):367–70. 8662526 10.1126/science.273.5273.367

[6] Lorange EA, Race BL, Sebbane F, Hinnebusch BJ. Poor vector competence of fleas and the evolution of hypervirulence in *Yersinia pestis* . J Inf Dis. 2005;191[11]:1907–12. 15871125 10.1086/429931

[7] Pujol C, Bliska JB. Turning *Yersinia* pathogenesis outside in: subversion of macrophage function by intracellular yersiniae. Clin Immunol. 2005;114[3]:216–26. 15721832 10.1016/j.clim.2004.07.013

[8] Spinner JL, Cundiff JA, Kobayashi SD. *Yersinia pestis* type III secretion system-dependent inhibition of human polymorphonuclear leukocyte function. Infect Immun. 2008;76[8]:3754–60. 10.1128/IAI.00385-08 18490459 PMC2493194

[9] Spinner JL, Winfree S, Starr T, Shannon JG, Nair V, Steele-Mortimer O, et al *Yersinia pestis* survival and replication within human neutrophil phagosomes and uptake of infected neutrophils by macrophages. Journal of leukocyte biology. 2013. Epub 2013/11/15. 10.1189/jlb.1112551 PMC3923079 24227798

[10] Janssen WA, Surgalla MJ. Plague bacillus: survival within host phagocytes. Science. 1969;163(3870):950–2. Epub 1969/02/28. 5763880 10.1126/science.163.3870.950

[11] Marketon MM, DePaolo RW, DeBord KL, Jabri B, Schneewind O. Plague bacteria target immune cells during infection. Science. 2005;309(5741):1739–41. 16051750 10.1126/science.1114580 PMC3210820

[12] Bliska JB, Wang X, Viboud GI, Brodsky IE. Modulation of innate immune responses by *Yersinia* type III secretion system translocators and effectors. Cellular microbiology. 2013;15[10]:1622–31. Epub 2013/07/10. 10.1111/cmi.12164 23834311 PMC3788085

[13] Cornelis GR, Wolf-Watz H. The *Yersinia* Yop virulon: a bacterial system for subverting eukaryotic cells. Mol Microbiol. 1997;23[5]:861–7. 9076724 10.1046/j.1365-2958.1997.2731623.x

[14] Vadyvaloo V, Jarrett C, Sturdevant DE, Sebbane F, Hinnebusch BJ. Transit through the flea vector induces a pretransmission innate immunity resistance phenotype in *Yersinia pestis* . PLoS Pathog. 2010;6[2]:e10000783. 10.1371/journal.ppat.1000783 PMC2829055 20195507

[15] Cavanaugh DC. Specific effect of temperature upon transmission of the plague bacillus by the oriental rat flea, *Xenopsylla cheopis* . Am J Trop Med Hyg. 1971;20[2]:264–73. 5553266 10.4269/ajtmh.1971.20.264

[16] Shannon JG, Hasenkrug AM, Dorward DW, Nair V, Carmody AB, Hinnebusch BJ. *Yersinia pestis* subverts the dermal neutrophil response in a mouse model of bubonic plague. mBio. 2013;4[5]:e00170–13. Epub 2013/08/29. 10.1128/mBio.00170-13 23982068 PMC3760243

[17] Andersen JF, Hinnebusch BJ, Lucas DA, Conrads TP, Veenstra TD, Pham VM, et al An insight into the sialome of the oriental rat flea, *Xenopsylla cheopis* (Rots). BMC Genomics. 2007;8:102 17437641 10.1186/1471-2164-8-102 PMC1876217

[18] Peters NC, Egen JG, Secundino N, Debrabant A, Kimblin N, Kamhawi S, et al In vivo imaging reveals an essential role for neutrophils in leishmaniasis transmitted by sand flies. Science. 2008;321(5891):970–4. Epub 2008/08/16. 10.1126/science.1159194 18703742 PMC2606057

[19] Platt AM, Randolph GJ. Dendritic cell migration through the lymphatic vasculature to lymph nodes. Advances in immunology. 2013;120:51–68. Epub 2013/09/28. 10.1016/B978-0-12-417028-5.00002-8 24070380

[20] Lindquist RL, Shakhar G, Dudziak D, Wardemann H, Eisenreich T, Dustin ML, et al Visualizing dendritic cell networks in vivo. Nat Immunol. 2004;5[12]:1243–50. Epub 2004/11/16. 15543150 10.1038/ni1139

[21] Deoras PJ, Prasad RS. Feeding mechanism of Indian fleas *X*. *cheopis* (Roths) and *X*. *astia* (Roths). The Indian journal of medical research. 1967;55[10]:1041–50. Epub 1967/10/01. 5594375

[22] Lavoipierre MMJ, Hamachi M. An apparatus for observations on the feeding mechanism of the flea. Nature. 1961;192:998–9.

[23] Lammermann T, Afonso PV, Angermann BR, Wang JM, Kastenmuller W, Parent CA, et al Neutrophil swarms require LTB4 and integrins at sites of cell death in vivo. Nature. 2013;498(7454):371–5. Epub 2013/05/28. 10.1038/nature12175 23708969 PMC3879961

[24] Spinner JL, Carmody AB, Jarrett CO, Hinnebusch BJ. Role of *Yersinia pestis* toxin complex family proteins in resistance to phagocytosis by polymorphonuclear leukocytes. Infect Immun. 2013;81[11]:4041–52. Epub 2013/08/21. 10.1128/IAI.00648-13 23959716 PMC3811843

[25] Rebeil R, Jarrett CO, Driver JD, Ernst RK, Oyston PC, Hinnebusch BJ. Induction of the *Yersinia pestis* PhoP-PhoQ regulatory system in the flea and its role in producing a transmissible infection. J Bacteriol. 2013;195[9]:1920–30. Epub 2013/02/26. 10.1128/JB.02000-12 23435973 PMC3624595

[26] Vuong C, Voyich JM, Fischer ER, Braughton KR, Whitney AR, DeLeo FR, et al Polysaccharide intercellular adhesin (PIA) protects *Staphylococcus epidermidis* against major components of the human innate immune system. Cellular microbiology. 2004;6[3]:269–75. Epub 2004/02/07. 14764110 10.1046/j.1462-5822.2004.00367.x

[27] Hume DA. Applications of myeloid-specific promoters in transgenic mice support in vivo imaging and functional genomics but do not support the concept of distinct macrophage and dendritic cell lineages or roles in immunity. Journal of leukocyte biology. 2011;89[4]:525–38. Epub 2010/12/21. 10.1189/jlb.0810472 21169519

[28] Kawai T, Akira S. Toll-like receptors and their crosstalk with other innate receptors in infection and immunity. Immunity. 2011;34[5]:637–50. Epub 2011/05/28. 10.1016/j.immuni.2011.05.006 21616434

[29] Jawetz E, Meyer KF. The behaviour of virulent and avirulent *Pasteurella pestis* in normal and immune experimental animals. J Inf Dis. 1944;74:1–13.

[30] Sebbane F, Gardner D, Long D, Gowen BB, Hinnebusch BJ. Kinetics of disease progression and host response in a rat model of bubonic plague. Am J Pathol. 2005;166[5]:1427–39. 15855643 10.1016/S0002-9440(10)62360-7 PMC1606397

[31] Gonzalez RJ, Lane MC, Wagner NJ, Weening EH, Miller VL. Dissemination of a highly virulent pathogen: tracking the early events that define infection. PLoS Pathog. 2015;11[1]:e1004587 10.1371/journal.ppat.1004587 25611317 PMC4303270

[32] Faust N, Varas F, Kelly LM, Heck S, Graf T. Insertion of enhanced green fluorescent protein into the lysozyme gene creates mice with green fluorescent granulocytes and macrophages. Blood. 2000;96[2]:719–26. 10887140

