# Supplementary material for: Dermal Neutrophil, Macrophage and Dendritic Cell Responses to Yersinia pestis Transmitted by Fleas
Source: PLoS Pathog. 2015 Mar 17;11(3):e1004734. doi: 10.1371/journal.ppat.1004734 (PMC4363629; doi:10.1371/journal.ppat.1004734)
Supplement: S1 Table — The indicated number of Y. pestis pMcherry blocked fleas were exposed to the right ear of individual LysM-eGFP or CD11c-YFP mice for 50 min. These mice were then imaged by intravital microscopy for approx. 4 h. Mice were euthanized at ~5 h post-feeding and the right ear, right mandibular and parotid lymph nodes, and spleen were harvested, triturated and plated to determine Y. pestis CFU counts. The results of each individual experiment are shown. Data are sorted in descending order based on the total number of fleas that fed on each mouse. (DOCX) [file ppat.1004734.s001.docx]

| Mouse Strain | Ear | dLN | Spleen | Total  # of fleas | # of fleas that fed |
| --- | --- | --- | --- | --- | --- |
| LysGFP | 275 | 405 | 1350 | 7 | 7 |
| LysGFP | 275 | 870 | 280 | 6 | 6 |
| LysGFP | 185 | 50 | 5 | 5 | 5 |
| LysGFP | 20 | 0 | 1 | 6 | 5 |
| LysGFP | 5 | 15 | 19 | 6 | 5 |
| LysGFP | 550 | 270 | 77 | 8 | 5 |
| LysGFP | 815 | 500 | 163 | 6 | 5 |
| LysGFP | 2670 | 0 | 1600 | 6 | 4 |
| LysGFP | 0 | 0 | 0 | 6 | 4 |
| LysGFP | 385 | 370 | 1700 | 6 | 4 |
| LysGFP | 150 | 20 | 2 | 5 | 3 |
| LysGFP | 1920 | 0 | 971 | 5 | 3 |
| LysGFP | 0 | 0 | 0 | 3 | 2 |
| LysGFP | 25 | 0 | 0 | 2 | 2 |
| LysGFP | 0 | 0 | 0 | 2 | 2 |
| LysGFP | 575 | 600 | 0 | 2 | 2 |
| LysGFP | 160 | 95 | 0 | 5 | 2 |
| LysGFP | 0 | 0 | 0 | 4 | 2 |
| LysGFP | 70 | 25 | 4 | 6 | 2 |
| LysGFP | 3465 | 0 | 42 | 6 | 2 |
| LysGFP | 5 | 0 | 0 | 2 | 1 |
| LysGFP | 0 | 0 | 0 | 2 | 1 |
| LysGFP | 125 | 465 | 1 | 1 | 1 |
| LysGFP | 0 | 0 | 0 | 4 | 1 |
| LysGFP | 0 | 0 | 0 | 2 | 1 |
| LysGFP | 0 | 0 | 0 | 1 | 1 |
| CD11cYFP | 365 | 0 | 77 | 6 | 6 |
| CD11cYFP | 3660 | 300 | 458 | 6 | 5 |
| CD11cYFP | 200 | 20 | 36 | 6 | 5 |
| CD11cYFP | 330 | 185 | 22 | 6 | 5 |
| CD11cYFP | 151 | 0 | 4000 | 6 | 4 |
| CD11cYFP | 0 | 0 | 7 | 6 | 4 |
| CD11cYFP | 730 | 65 | 0 | 6 | 4 |
| CD11cYFP | 10 | 0 | 107 | 6 | 3 |
| CD11cYFP | 175 | 1000 | 14 | 6 | 3 |
| CD11cYFP | 0 | 0 | 35 | 6 | 2 |

**Table S1.** **CFU transmission data acquired after intravital microscopy experiments at ~5 h post-feeding.**
