# Supplementary figures and images for: Dermal Neutrophil, Macrophage and Dendritic Cell Responses to Yersinia pestis Transmitted by Fleas
Source: PLoS Pathog. 2015 Mar 17;11(3):e1004734. doi: 10.1371/journal.ppat.1004734 (PMC4363629; doi:10.1371/journal.ppat.1004734)

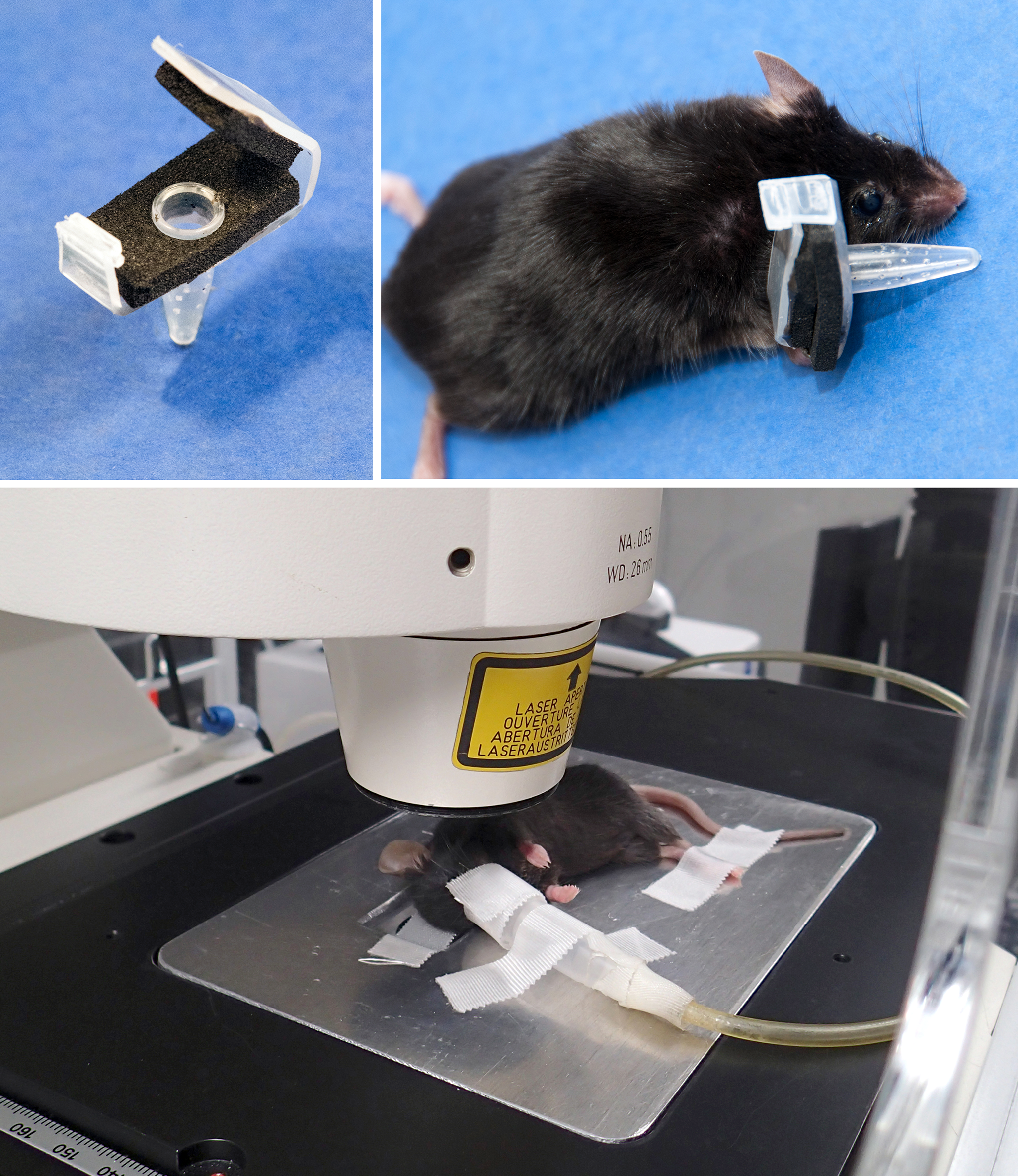

Supplement: S1 Fig — (TIFF) [file ppat.1004734.s002.tiff]

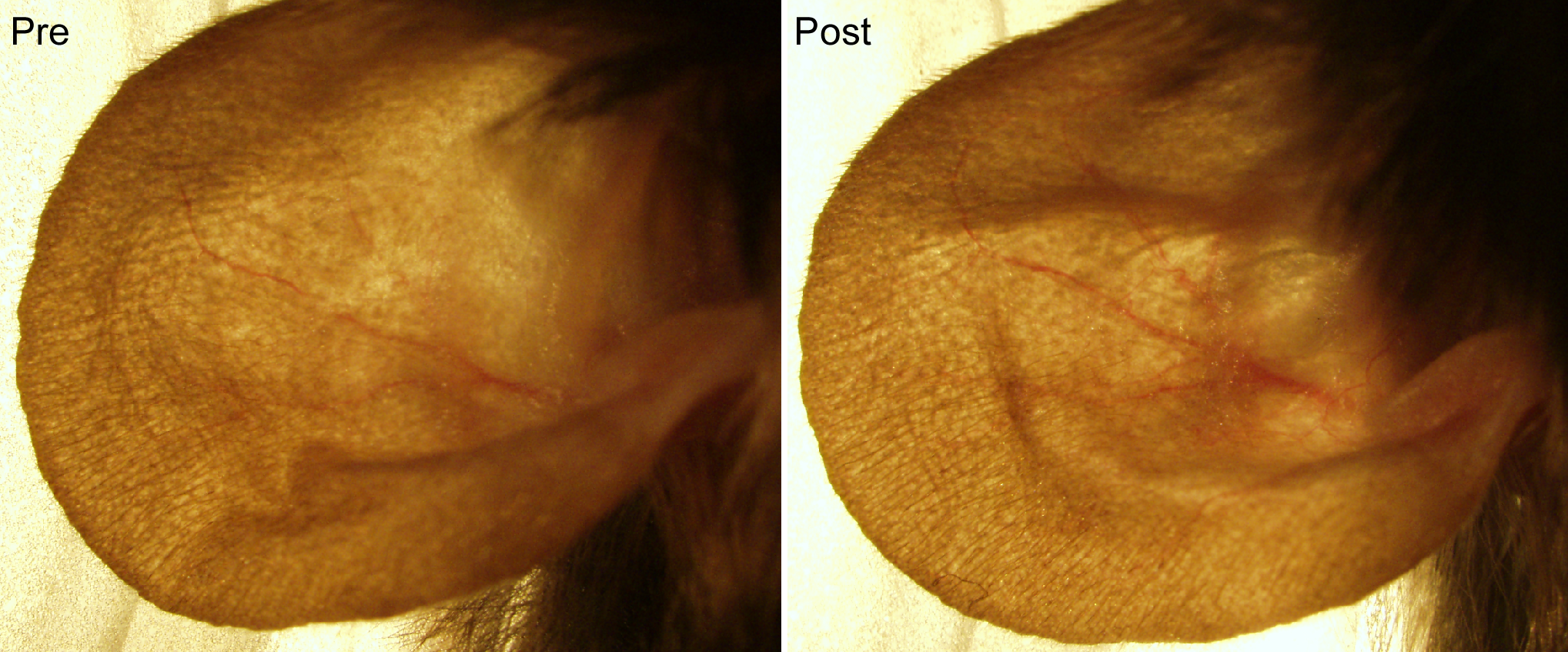

Supplement: S2 Fig — Dissecting microscope images of a mouse ear before (Pre) and after (Post) being fed upon by 3 uninfected fleas for 10 min. No obvious flea bite sites can be seen, but blood vessel dilation in response to uninfected flea feeding is apparent. (TIFF) [file ppat.1004734.s003.tiff]
